# Assessment of Intraday Variations in Skin Indentation Resistance

**DOI:** 10.7759/cureus.65590

**Published:** 2024-07-28

**Authors:** Harvey N Mayrovitz

**Affiliations:** 1 Medical Education, Nova Southeastern University Dr. Kiran C. Patel College of Allopathic Medicine, Davie, USA

**Keywords:** skinfibrometer, skin compression, skin induration, skin fibrosis, skin biophysical properties, skin mechanical properties, skin elasticity

## Abstract

Introduction: Information about the mechanical properties of skin and their changes with age and other conditions is important to help characterize skin physiology and pathological changes. One method to obtain this information is to measure the force required to indent the skin to a specified indentation depth (FORCE). This process measures the tissue’s resistance to indentation or its compressibility and is related to the tissue's elastic modulus. Since such measurements are made in clinical and other settings at various times of day (TOD), it is useful to estimate the extent of intraday variations in FORCE that may be expected. This report focuses on this issue.

Method: FORCE was self-measured on the volar forearm, 5 cm distal to the antecubital fossa, every two hours from 08:00 to 24:00 hours on two consecutive days by 12 medical students (six females and six males) who were trained in the measurement process using an indentation device (SkinFibroMeter). Variability in FORCE versus TOD was analyzed using the nonparametric Friedman test and differences between genders by the nonparametric Wilcoxon test. Differences between the first day (day 1) and the second day (day 2) were tested at each TOD. The whole-body fat percentage (FAT%) and water percentage (H_2_O%) were determined for each participant via bioimpedance measurements at 50 KHz.

Results: The age and BMI of the combined group (mean ± SD) were 24.5 ± 1.5 years and 23.2 ± 3.3 kg/m^2.^ The overall average FORCE (mean ± SD) for the day over the 16 hours was 84.1 ± 22.7 mN and for day 2, it was 83.4 ± 28.5 mN with no significant difference between day 1 and day 2. For females, the overall two-day average FORCE (mean ± SD) over the 16 hours was 81.8 ± 20.3 mN and for males, it was 85.7 ± 30.1 mN with no significant difference between them (p = 0.271). Overall, there was no statistically significant difference in FORCE among TOD (p = 0.568). FORCE was not correlated with either FAT%, HTO%, or BMI.

Conclusion: The findings indicate no statistically significant variation in indentation force in females, males, or combined concerning the TOD of the measurement or differences between consecutive days at corresponding times. This suggests that whether such measurements are done in a research setting or within a clinic, they can be done at various TOD with minimal expected variation for a given subject. However, an extension of these findings to persons with skin conditions or ages not herein evaluated must await further study.

## Introduction

Information about the mechanical properties of skin and their changes with age and other conditions is useful to help characterize skin physiology [1-5] and pathological changes [6-11]. One method to obtain this information is to measure the force required to indent the skin to a specified indentation depth. This process measures the tissue’s resistance to indentation or its compressibility [12-14]. Various devices have been developed and used to measure elastic or viscoelastic skin properties with varying degrees of complexity [15-18]. However, from the point of view of clinical utility, it is important to have an uncomplicated yet accurate assessment method. One such method is provided by a handheld device that employs a 2.5 mm diameter indentor that upon contact with the skin penetrates 1.3 mm and automatically records the force required for this indentation [12-14]. From a fundamental analytical point of view, the relationship between indentation force (FORCE) and indentation depth (d) for soft tissues with an overall soft tissue thickness H can be expressed as $\text{FORCE} = \delta \times E \times D \times \left( \frac{K}{1 - v^2} \right)$ in which E is the effective elastic modulus of the tissue, D the indentor diameter, v the Poisson’s ratio for the tissue and a factor K, that depends on the ratio of both δ/H and D/H. Thus, the measured force depends directly on penetration depth and importantly on the soft tissue elastic modulus [19-21]. Since such measurements are made in clinical and other settings at various times of day (TOD), it would be useful to estimate the extent of intraday variations in force values that may be expected. This report focuses on this issue by evaluating the indentation force measured every two hours from 08:00 to 24:00 hours on two consecutive days in 12 healthy young adults.

## Materials and methods

Methods

Subjects

Twelve young healthy adult medical students (six males and six females) participated in this self-measurement research approved by the Nova Southeastern University Institutional Review Board (#2021-505). To be a part of the study, subjects needed to agree to be trained and certified in all measurement methods and be willing and able to do these self-measurements at two-hour intervals from 08:00 to 24:00 hours on two consecutive days of their choice. Exclusions to participation were any skin conditions or open wounds affecting forearm skin which was to be the site of the measurements.

Measurements

**Figure 1:**
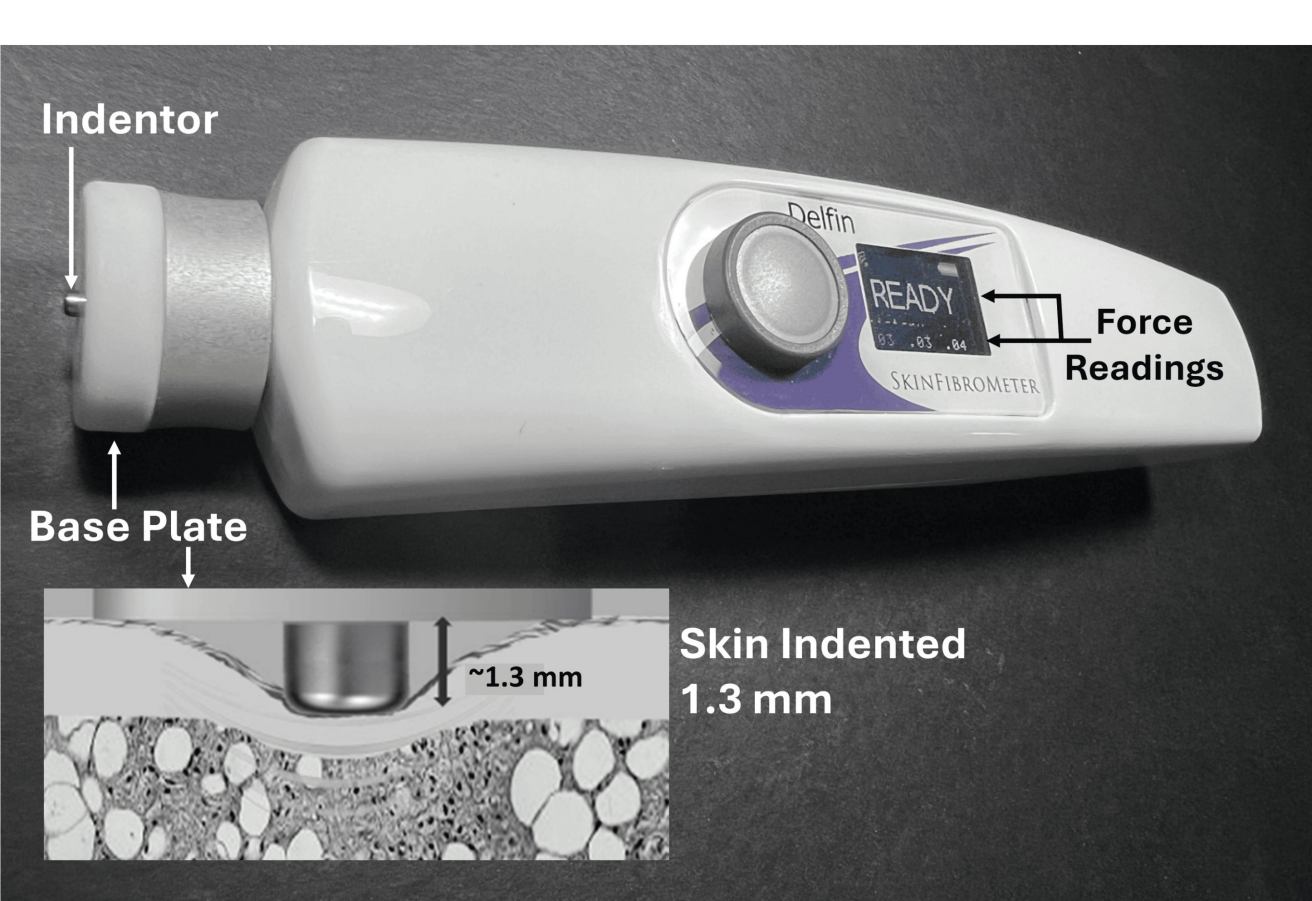
Device for measuring indentation force The force required to indent the skin 1.3 mm is registered on the display as the average of five rapid indentations. The indentor diameter is approximately two mm and the base plate, which remains in contact with the skin, has a diameter of 22 mm. Credit: The figure is courtesy of Dr. HN Mayrovitz.

Skin indentation force was measured with the SkinFibroMeter (Delfin Technologies, Kuopio, Finland) as shown in Figure 1. The indentation force in mN required to indent skin to 1.3 mm (FORCE) was determined. In use, skin is lightly touched whereupon a small indenter approximately 2.0 mm in diameter is caused to deform skin inwardly with the resultant force recorded and displayed on a window on the front of the device. The device is equipped with internal sensors that accept measurements only within prescribed limits of the force and velocity of the skin contact. This means that if an applied force is too large or applied too slowly or rapidly, the software contained within the device prompts to repeat the measurement until it is within the set limitations of the device [4]. A single recorded value is obtained as the average of five acceptable sequential measurements made rapidly in succession. The time to make these five sequential measurements at a single site is about 5 seconds. In this study, this sequence was repeated three times, and an average value was obtained. Skin temperature (TSK) was measured once at the same site using an infrared thermometer (Model DX501-RS, Exergen, Watertown Main, USA) with a stated repeatability of ±0.1^o^C. Measurements were done while the participants were seated with their nondominant arm relaxed and comfortably resting palm up on a flat surface, approximately at heart level. On a day prior to the start of skin measurements, each participant’s whole-body fat and water percentages (FAT% and H20%) were measured once using bioimpedance at 50 KHz (InnerScan Body Composition Monitor, Tanita model BC558, The Competitive Edge, Vancouver, USA). Body composition measurements were made with participants barefooted while standing on a scale for about 10 seconds while gripping handle electrodes. Room temperature (TRM) and relative humidity (RH) were measured (Fluke Model 971, Everett, USA) with a stated accuracy of ±0.1^o^C for TRM and ±2.5% for RH.

Initial procedures

Before starting self-measurements, each participant was trained and evaluated in the proper use of the SkinFibroMeter by the author during a dedicated training session. During that session, but following their training, each participant performed a series of measurements that replicated what they would do during their self-measurement protocol. This sequence of measurements was observed for proper technique and corrected if needed, and a second sequence was carried out. As an index of the adequacy of the self-measurer, the degree to which their measurements matched those done by the author on their arm needed to be within 5%. No participant required any further corrections. After this training and validation session the total body weight, FAT%, and H2O% were measured.

Self-measuring procedure

The self-measurer made their measurements on two consecutive days, usually the weekend, while at home for the entire day. No lotions, creams, or other substances were permitted to be applied to the forearm on measurement days. On the first measurement day (day 1), a point on the nondominant volar forearm located 5 cm distal to the antecubital fossa was marked with a surgical pen. This would be the site of all skin measurements. This same mark remained visible and was used on both days. The self-measurements started with the triplicate indentation force measurements and then the single skin measurement. After this, the room temperature and RH were recorded. These measurements were repeated every two hours starting at 08:00 hours and continuing to and including 24:00 hours. This constituted nine sequential measurement sets over 16 hours. During these two days, the measurer’s activities were confined to normal ones consisting of studying, watching TV, listening to music, and at times eating and drinking non-caffeinated beverages. No vigorous activity was permitted nor was washing the forearm permitted. The entire procedure was replicated the next day (day 2).

Analysis

The triplicate values of FORCE for each of the nine daily measurement times were averaged to yield one value for each measurement time. Tests for normality via the Shapiro-Wilk test at each measurement time indicated that normality could not be assumed for any time for either of the two days. Thus, statistics were based on nonparametric tests. To test for potential differences in FORCE values among the nine measurement times, the nonparametric Friedman test was used. Values for each parameter at each time were compared between day 1 and day 2 values using the non-parametric Mann-Whitney test. Tests for differences between genders were based on the Wilcoxon test. All statistical tests were done using SPSS for Windows, Version 16 (Released 2007; SPSS Inc., Chicago, USA). To evaluate if body habitus parameters (BMI, FAT%, H20%) impacted FORCE, the per-person time average of FORCE was determined as the average over all nine measured times for both days and the correlation among the parameters was determined.

## Results

Participant characteristics

Male and female participants had similar ages that ranged from 20 to 28 years with a mean ± SD of 24.5 ± 1.5 years for the entire group (N = 12). Males compared to females had a greater height (178.5 ± 6.3 vs. 164.8 ± 6.2 cm, p = 0.003 and weight 82.3 ± 7.5 vs. 60.4 ± 9.2 kg, p = 0.002) but there was no statistical difference in body mass index for male and females which were, respectively, 23.6 ± 3.7 vs. 22.8 ± 3.0 kg/m^2^, p = 0.518. However, males had less FAT% (21.2 ± 3.0 vs 31.4 ± 4.8, p < 0.01) and greater H_2_O% (61.4 ± 2.8 vs. 50.1 ± 3.2, p < 0.01).

Temperature and humidity variation among TOD

Tests for significant differences among times using the nonparametric Friedman test failed to detect a significant difference in TSK, TRM, or RH among TOD for either day 1 or day 2 measurements. They showed no significant trends over the 16-hour measurement interval. Averaging values over the nine measurement times for both days yielded an overall average ± SD for TSKAVG, TRMAVG, and RHAVG of 32.1 ± 1.1 ^o^C, 23.4 ± 1.8 ^o^C, and 52.0 ± 4.8%, respectively.

Inter-day indentation force comparisons

**Figure 2:**
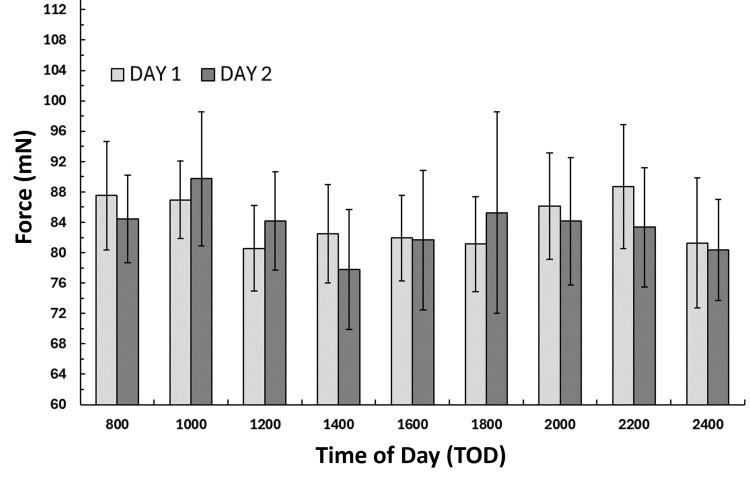
Inter-day indentation force Values are the mean indentation force at each time of day for the 12 participants and the error bars are the standard error of the mean (SEM). Forces did not significantly differ between days at any time of day.

FORCE at each measurement time was compared between day 1 and day 2 values using the non-parametric Wilcoxon test. The results showed no significant difference between day 1 and day 2 values at corresponding times. The FORCE values for day 1 and day 2 are shown in Figure 2. The overall average FORCE (mean ± SD) for day 1 over the 16 hours was 84.1 ± 22.7 mN and for day 2, it was 83.4 ± 28.5 mN with no significant difference between day 1 and day 2. The largest percentage difference between day 1 and day 2 was 1.56% at 22:00 hours and the least difference was 0.08% at 16:00 hours. For these calculations, the percentage difference was the day 1 - day 2 difference divided by the average of day 1 and day 2 values.

Indentation force by gender

**Figure 3:**
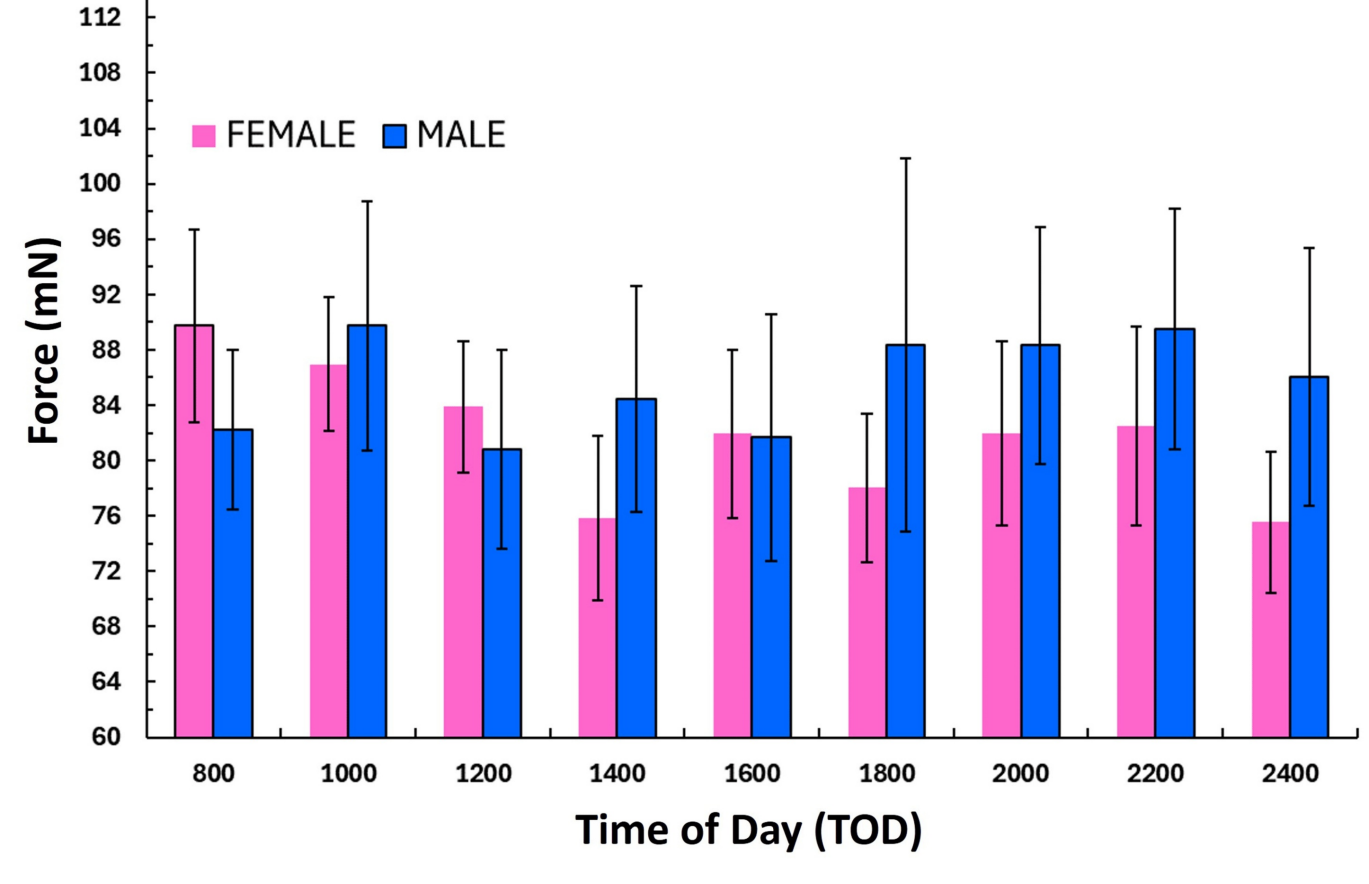
Female versus male indentation forces Values are the mean indentation force at each time of day for the six females and six males. The error bars are the standard error of the mean (SEM). Forces did not significantly differ between the sexes at any time of day.

FORCE at each measurement time was compared between female and male values using the non-parametric Mann-Whitney test. The results showed no significant difference between female and male values at corresponding times. The FORCE values for females and males are shown in Figure 3 for each TOD. For females, the overall two-day average FORCE (mean ± SD) over the 16 hours was 81.8 ± 20.3 mN and for males, it was 85.7 ± 30.1 mN with no significant difference between them (p = 0.271). The largest percentage difference between genders was -3.25% at 24:00 hours and the least difference was also 0.08% at 16:00 hours. For these calculations, the percentage difference was the female-male difference divided by their average.

Indentation force by TOD

**Figure 4:**
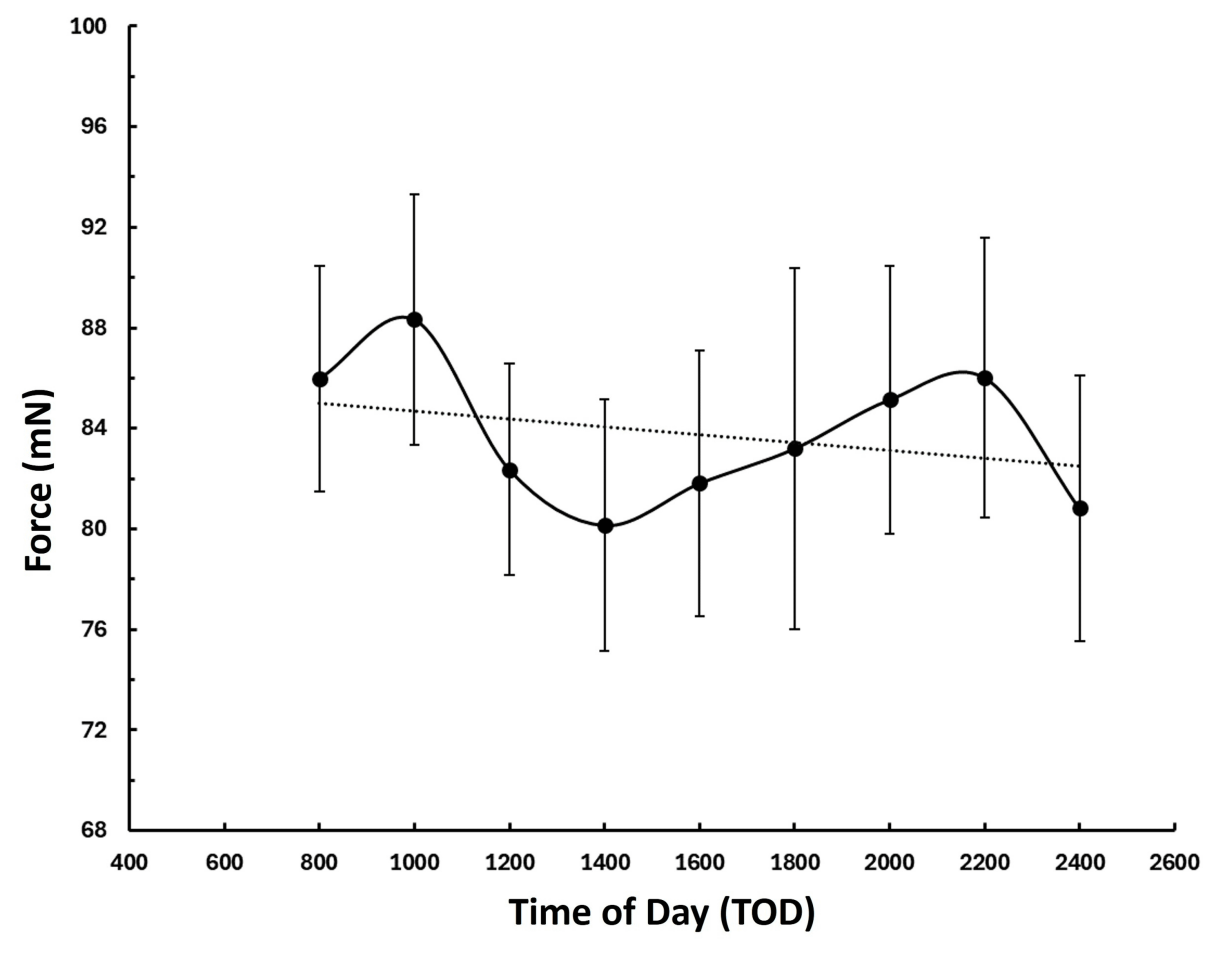
Indentation force by time of day Points are the indentation force averages of both days at each TOD for the 12 participants. Error bars are the standard error of the mean (SEM). The dotted line is the linear regression line. The apparent peaks of the mean at 10:00 and 22:00 are not significantly different from the apparent minimum at 14:00 hours. The regression is also not significant (p = 0.409).

Figure 4 shows the temporal pattern for the mean indentation force ± SEM as a function of TOD based on data from both days. The result of the nonparametric Friedman test indicates no statistically significant difference in FORCE among TOD (p = 0.568). The temporal pattern appears to suggest the presence of a mean value maximum at 10:00 hours of (mean ± SD) 88.3 ± 24.4 and a minimum at 14:00 hours of 80.1 ± 24.6. This would represent an overall percentage difference of about 2.5%. However, these mean values lie within the SEM and are not statistically different (p = 0.139). The linear regression of FORCE versus TOD indicates no significant temporal trend over the 16 hours (p = 0.409).

Indentation force dependence on body habitus parameters

There was no statistically significant correlation between FORCE and a participant’s BMI, FAT%, or H2O% with corresponding significance levels being 0.935, 0.670, and 0.687, respectively.

## Discussion

Measurements of the skin’s mechanical properties have been carried out for various purposes. These include ways to help characterize skin aging patterns in different types of skin [22-25], to evaluate skin conditions such as fibrosis [11,26], to characterize some lymphedema features [27,28], to compare mechanical properties of healthy versus lymphedematous skin [29], and to evaluate skin changes after treatment procedures [8,26]. Such investigations have been carried out with a variety of methods including those that rely on stretching the skin to determine its mechanical features and those that use skin indentation for that purpose. The present study has focused on the use of skin indentation given its potential to estimate skin’s elastic modulus and its use to assess skin induration and skin fibrosis. The device used in the present study has been shown to be useful and reliable for these purposes [11,13,14]. What had not been investigated was the extent of variability of the measurements concerning the TOD such measurements were made and were the primary focus of the present study.

The present study's main findings indicate no statistically significant temporal variation in the indentation force over TOD and no correlation between body habitus parameters and the magnitude of the indentation force. This finding is important since such assessments, whether done in a research setting or within a clinic, can be done at various TOD. However, because of the relatively small number of subjects included in the study, it may be that the inclusion of a larger number of subjects may very well uncover some consistent variations not herein detected.

It is also observed that there is a large variance in indentation force among subjects and that this fact combined with the number of participants may have masked the presence of a “real” temporal variation as demonstrated by the pattern shown in Figure 4. Thus, the present results cannot rule out the possibility of some sort of diurnal rhythm, which would need to be studied with a larger group. From the present data, however, the variation between the maximum observed at 10:00 and the minimum observed at 14:00 hours amounts to only 2.5% and is not likely to be of clinical relevance.

Study limitations

One limitation to be considered is that the data obtained is based on self-measurements done by multiple persons. Although each participant was well trained and certified in the measurement and protocol process by the author, this does not guarantee that, when not observed, errors may occur. However, the consistency of the overall data among all participants, which was carefully reviewed, indicates that any deviations would have been small and limited in overall effect.

Another limitation, as noted, is the number of participants and their demographics. The present findings apply specifically to the young adult healthy population herein studied and potential generalizations to either older populations or persons with any skin condition or greater variability in BMI must await further investigation. Such temporal studies could use the present results as baseline indicators.

## Conclusions

The findings indicate no statistically significant variation in indentation force in females, males, or combined concerning the TOD of the measurement or differences between consecutive days at corresponding times. This suggests that whether such measurements are done in a research setting or within a clinic, they can be done at various TOD with minimal expected variation for a given subject. However, an extension of these findings to persons with skin conditions or ages not herein evaluated must await further study. It should also be noted that because of the relatively small number of subjects included in the study, it may be that the inclusion of a larger number of subjects may uncover some consistent variations not herein detected.
